# Real-world evidence on tagraxofusp for blastic plasmacytoid dendritic cell neoplasm – collected cases from a single center and case reports

**DOI:** 10.3389/fonc.2024.1384172

**Published:** 2024-04-11

**Authors:** Philipp Faustmann, Jan C. Schroeder, Lucas Mix, Lennart Harland, Andreas Riedel, Wichard Vogel, Claudia Lengerke, Stefan Wirths

**Affiliations:** Department for Internal Medicine 2, Hematology, Oncology, Clinical Immunology and Rheumatology, University Hospital Tuebingen, Tuebingen, Germany

**Keywords:** BPDCN, tagraxofusp, orphan disease, hematology, targeted therapy, first in class therapy

## Abstract

**Introduction:**

Blastic plasmacytoid dendritic cell neoplasia (BPDCN) is a rare, aggressive hematologic malignancy. Until recently, the only curative treatment consisted of intensive chemotherapy, followed by hematopoietic cell transplantation (HCT) in eligible adult cases. Tagraxofusp, a CD123-targeted protein-drug conjugate and the first approved targeted treatment for BPDCN, might enhance outcomes especially in patients not eligible for intensive therapies.

**Methods:**

Here, we report real-world outcomes of five male patients with a median age of 79 years who received tagraxofusp as first-line treatment for BPDCN.

**Results:**

Tagraxofusp was found to be well-tolerated in this elderly cohort, with only one patient requiring discontinuation. Three patients responded to the treatment (two patients achieved a CR and one patient achieved a partial response), of which two subsequently underwent allogeneic (allo) HCT. One patient is alive and well after ≥ 4 years after alloHCT, and one patient shows sustained CR after now 13 cycles of tagraxofusp. The other three patients died of progressive disease 4-11 months after initiation of treatment.

**Discussion:**

In line with results from 13 published cases outside clinical trials in the literature, sustained responses were associated with CR after tagraxofusp treatment and subsequent alloHCT. Our results provide real-world evidence for safety and efficacy of tagraxofusp as first-line treatment for BPDCN.

## 1 Introduction

Blastic plasmacytoid dendritic cell neoplasia (BPDCN) is a rare hematologic disease with a poor outcome (1). With a reported incidence of 0.04 cases per 100.000 in the US, it represents less than 1% of all hematologic malignancies. The median age of onset of the adult variant is around 70 years and men are more frequently affected (2). The typical presentation consists of characteristic skin lesions as well as affection as leukemia or of lymph nodes, liver, spleen and, less frequently, other extramedullary sites such as the central nervous system (CNS). CNS involvement at diagnosis has been reported at varying frequencies with 2% to 13% and is likely under-appreciated due to a lack of standardized CNS testing in clinical practice (3–10). Mutations in genes associated with myeloid diseases/clonal hematopoiesis of indeterminate potential (CHIP), such as DNA methylation, signal transduction and RNA splicing genes, are often found (11–14). The immunophenotype is characterized as CD4+/CD56+/CD123+/HLA-DR+/TCL1+/CD303+, but markers from myeloid and lymphoid lineages may also be present (7, 11, 15, 16). Interestingly, BPDCN is frequently found in conjunction with other myeloid neoplasms such as chronic myelomonocytic leukemia (CMML), myelodysplastic syndrome (MDS), or acute myeloid leukemia (AML) (11, 17, 18).

The median overall survival (OS) of adult BPDCN ranges between 7 to 24 months in retrospective cohorts (8, 19, 20). Historically, BPDCN was treated with intensive chemotherapy analogous to treatment protocols for AML, acute lymphoblastic leukemia (ALL), or aggressive lymphoma. Intensive chemotherapy was associated with a 2-year progression-free survival (PFS) of 45% in a cohort of 59 patients (21), and ALL regimens yielded superior results in retrospective analyses. In contrast, a large retrospective analysis indicated a median PFS of only 10 months, irrespective of treatment (8). Consolidation with allogeneic hematopoietic cell transplantation (alloHCT) enables long-term median OS rates up to 6.6 years, thus prolonging median OS by 3- to 5-fold (21–25). Myeloablative conditioning (MAC) with total body irradiation (TBI) was significantly superior compared to other forms of conditioning, although MAC was more often used in younger patients. For patients ineligible for MAC with TBI, autologous (auto)HCT seems to be an option (22, 25, 26). However, intensive chemotherapy, alloHCT and autoHCT can only be performed in a fraction of BPDCN patients because of either advanced age, frailty or comorbidities.

In 2018, treatment of BPDCN was revolutionized by the introduction of tagraxofusp (SL-401) as the first targeted and disease-specific agent (27). Overexpression of interleukin-3 receptor subunit alpha or CD123 is a long-recognized hallmark of BPDCN (28–31). Tagraxofusp, a fusion protein of diphtheria toxin and interleukin 3, has shown cytotoxic activity in BPDCN *in vitro* and *in vivo* models (32) due to internalization of the toxin payload in CD123-positive neoplastic cells.

An early phase 1-2 study of 11 BPDCN patients showed an overall response rate of 78% and reasonable tolerability of treatment with tagraxofusp. Capillary leak syndrome (CLS), a vascular toxicity resulting in edema, hypervolemia and cardiovascular events, was identified as a tagraxofusp-specific adverse effect in a few patients (33). Due to these promising results, the open-label, multicenter, multistaged trial STML-401-0114 was conducted and enrolled 47 untreated or relapsed BPDCN patients for treatment with tagraxofusp (34). Treatment was applied at 7 to 12 µg per kg of body weight for five consecutive days and continued in 21-day cycles until progression or intolerable side effects. Among the eligibility criteria, patients needed to demonstrate good performance status (Eastern Cooperative Oncology Group (ECOG) of 0-2), and normal cardiopulmonary, renal, and hepatic function as well as normal albumin to minimize the risk for CLS development. The overall response rate of previously untreated patients was 90%, with complete response (CR) observed in 72%. 45% of these patients subsequently received alloHCT and 2-year OS was 52%. In relapsed patients, the overall response rate was 67%, and median survival was 8.5 months. Grade 3 or higher adverse events were reported in 81%, the most common being increased liver enzymes, thrombocytopenia, and CLS. Of 47 patients, one patient died of CLS and one CLS grade 4 event occurred, leading to the recognition of CLS as a key toxicity and the necessity of attentive monitoring and early intervention.

Long-term results of an expanded cohort of the STML-401-0114 trial recently indicated an overall response rate of 75%, a median CR duration of 24.9 months, and a median OS of 15.8 months after four treatment cycles, with overall good safety profile (35).

Given the low incidence of BPDCN, awareness regarding toxicity, and cost issues, real-world data reporting on the safety and efficacy of tagraxofusp for the treatment of BPDCN are clearly needed. Therefore, we report here outcomes and clinical course of five patients who received tagraxofusp as first-line treatment for BPDCN at our center.

## 2 Materials and methods

All patients diagnosed with BPDCN and treated with tagraxofusp at our center between 2019 and 2023 were included. Diagnosis was confirmed by reference pathology. Tagraxofusp was administered intravenously at a dose of 12 µg/kg for five consecutive days in 21-day-cycles, unless adverse events made an interruption necessary. Patients routinely received the first cycle of treatment in the inpatient setting, and were monitored for the development of CLS by screening for hypoalbuminemia, weight gain, edema, or dyspnea, throughout treatment. Patients were routinely screened for CNS involvement by cerebrospinal fluid (CSF) collection, and partly received intrathecal chemotherapy with 12 mg or 15 mg methotrexate, 40 mg cytarabine and 4 mg dexamethasone or methotrexate only.

Demographic and clinicopathological variables were collected, including age, sex, medical comorbidities, performance status, blood count and lactate dehydrogenase, blast counts in peripheral blood (PB) and bone marrow (BM), immunophenotype, cytogenetic and molecular genetic aberrations, CNS involvement, line of treatment, treatment duration, post-remission and relapse treatments. For outcome measures, we analyzed best treatment response using the response criteria for BPDCN as published previously (34), duration of response, OS as defined by the time from treatment initiation to death of any cause, relapse, cause of death, and type and severity of adverse events according to the Common Terminology Criteria for Adverse Events (CTCAE) criteria. Data lock was at January 4, 2024. This analysis was reviewed and approved by our institutional review board (#623/2023BO2).

Descriptive statistics used absolute frequencies and percentages for categorical variables and median, arithmetic mean, range and standard deviation for continuous variables. Individual patient histories are depicted in a swimmer plot. The probability of OS was analyzed using the Kaplan-Meier estimator.

## 3 Results

### 3.1 Patient characteristics

**Figure 1:**
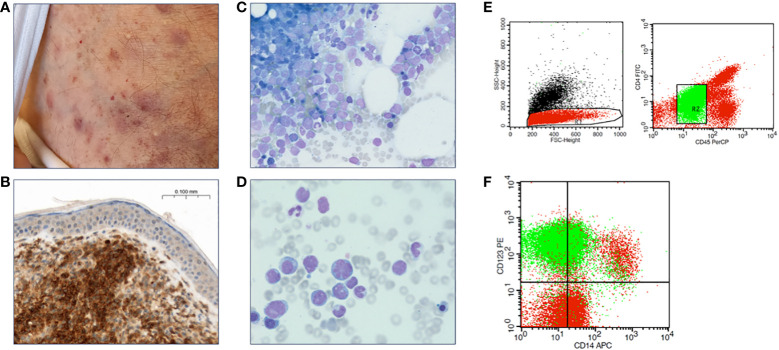
Diagnostic features of BPDCN. **(A)** Contusiform skin efflorescences characteristic for BPDCN. **(B)** Skin biopsy with CD123 immunohistochemical staining reveals infiltration by BPDCN. **(C, D)** Bone marrow cytology, Pappenheim staining. **(E)** Flow cytometry of a bone marrow aspirate, gating strategy. **(F)** Bone marrow BPDCN population in green.

Five patients, all male, with a median age of 79 years (range 63-84) were diagnosed with BPDCN at our institution (**Table 1**). All patients presented with systemic disease, including both skin and BM involvement (**Figure 1**). Other sites of involvement were the CNS in at least one patient (and one inconclusive result with no BPDCN cells cytologically in the cerebrospinal fluid, but low infiltration in flow cytometry, which could be an artefact from the procedure), the spleen in two patients, and lymph nodes, liver, and bone in one patient, respectively.

**Table 1:** Patient characteristics.

Patients				Disease parameters							Laboratory results at diagnosis					
#	Sex	Age	HCT-CI	Skin	BM	CNS	Other	Immunophenotype	Cytogenetic abnormalities	Molecular abnormalities (VAF)	LDH in U/l	Thrombocytes in G/l	HB in g/dl	Leukocytes in G/l	Blast count BM	Blast count PB
1	m	82	7	Yes	Yes	Putative	–	CD123+, CD4+, CD56+, TdT+	del12p13, del13q14	3x TET2 (34-50%), SRSF2 (19%), SH2B3 (48%), KIT (51%)	169	46	11,8	4	40%	31%
2	m	68	0	Yes	Yes	–	Putative lymph nodes	CD123+, CD4+, CD56+	–	ASXL1 (46%), 4x TET2 (7-36%)	151	181	12,7	4,9	4%	0%
3	m	84	5	Yes	Yes	–	Spleen	CD123+, CD4+, CD56+	del12p13	ASXL1 (38%), NRAS (35%), U2AF1 (43%), 2x SETBP1 (2%)	2319	60	10,1	83	84%	88%
4	m	63	7	Yes	Yes	Yes	Bone, spleen, liver	CD123+, CD4+, CD56+, TCL1+	del6q21, del6q23, del17p, CDKN1B-Del.	–	608	60	14,3	10,5	64%	22%
5	m	79	4	Yes	Yes	–	–	CD123+, CD4+, CD56+, TCL1+, BCL2+, CD43+	–	2x TET2 (39-50%), 3x CBL (8-39%), KRAS (7%), NRAS (5%), SH2B3 (11%)	137	136	11,7	11,3	25%	0%

BM, bone marrow; CNS, central nervous system; HB, hemoglobin; HCT-CI, hematopoietic cell transplantation comorbidity index; LDH, lactate dehydrogenase; PB, peripheral blood; VAF, variant allele frequency.

Two patients were additionally diagnosed with CMML, of whom one also showed systemic mastocytosis. The median HCT-comorbidity index (HCT-CI) was 5 (range 0-7) and the median ECOG score was 1 (range 0-1). Among the documented comorbidities, two patients had a history of prior malignancy (one patient, colon and prostate cancer; one patient, squamous cell carcinoma of the skin); two patients had significant cardiopulmonary disease (one patient, coronary artery disease, atrial fibrillation, chronic obstructive pulmonary disease; one patient, coronary artery disease, valvular heart disease); and one patient had a history of prior hepatitis B.

All patients exhibited expression of markers previously reported for BPDCN, including positivity for CD123, CD4, and CD56. The karyotype was normal in all patients examined, but molecular cytogenetic analysis revealed among others a del17p in one patient. Among the detected genetic variants, TET2 and ASXL1 mutations were reported in three and two patients, respectively (**Table 1**).

### 3.2 Treatment

All patients received tagraxofusp as first-line treatment, at a median time from diagnosis of 92 days (range 7-262). One patient had received azacitidine (Aza) prior to tagraxofusp for the treatment of CMML diagnosed at the same time as BPDCN, without any response of the latter. A median of five (range 1-13) cycles with a median treatment duration of 26 days (range 3-388) were administered. Two patients received cytoreductive chemotherapy in analogy to the GMALL treatment protocol (dexamethasone 10 mg/m² for five days and cyclophosphamide 200 mg/m² for three days) before initiation of tagraxofusp. Three patients received tagraxofusp before approval in the European Union, the other two patients were treated in-label.

Four of the five patients were screened for CNS involvement by CSF examination. Four patients, one of whom had proven asymptomatic CNS involvement, received intrathecal chemotherapy with methotrexate, cytarabine and dexamethasone (three patients) or methotrexate only (one patient). The patient with positive CNS involvement became negative after intrathecal therapy and did not relapse in the CNS. None of these patients developed documented CNS involvement during follow-up. CNS screening was not routinely performed in one of the five patients, and patients were not routinely tested for asymptomatic CNS involvement in the follow-up.

Two patients received alloHCT for consolidation, one in CR and one in partial remission (PR). Both received reduced-intensity conditioning with 8 Gray TBI and fludarabine 120 mg/m². The donor was HLA-matched (10/10) unrelated in both cases and transplant-related morbidity was low, with one patient developing mild bleeding, mucositis and systemic infection, but no higher-grade toxicities. None of them developed clinically significant graft-versus-host disease.

### 3.3 Safety and efficacy

Three patients developed CLS, with a maximum grade of 3 in one patient, leading to discontinuation of tagraxofusp after three doses. CLS was treated with loop diuretics and albumin substitution according to the recommendations. One patient developed acute kidney injury and one patient developed non-ST-elevation myocardial infarction with questionable association to tagraxofusp treatment due to a history of coronary artery disease. One patient had no treatment-related complications. Three patients developed hepatic toxicity (maximum of grade 3 in two patients) with elevated glutamate oxaloacetate transaminase (GOT) and glutamate pyruvate transaminase (GPT), and two patients developed thrombocytopenia (maximum of grade 4). All patients recovered from the respective toxicities.

**Figure 2:**
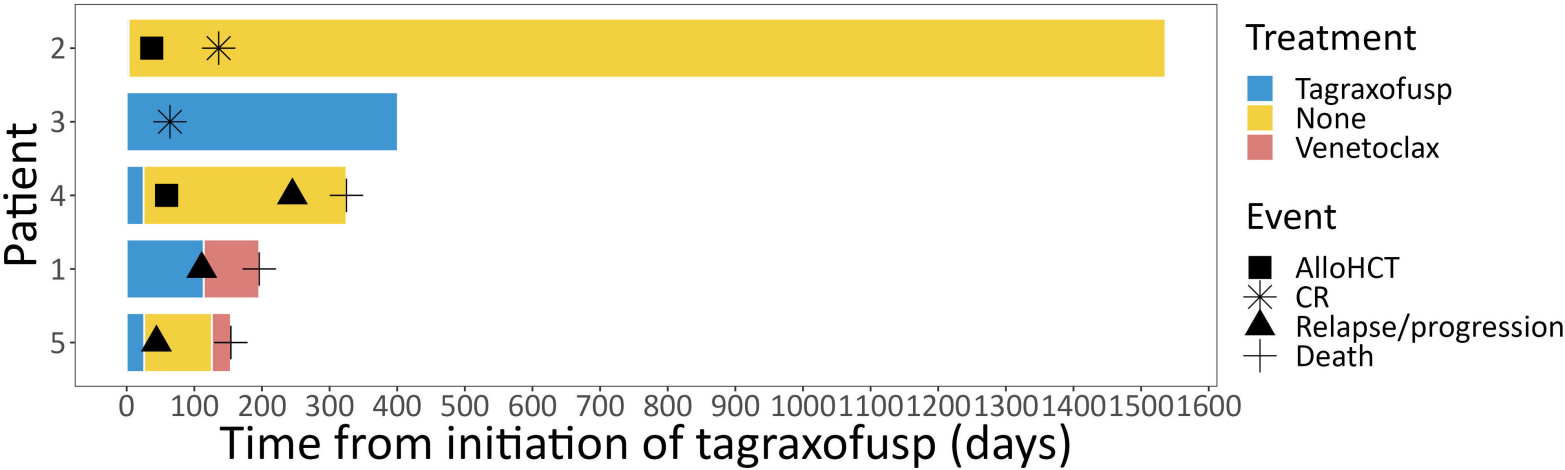
Course of treatment. Individual course of treatment from initiation of tagraxofusp depicted for each individual patient as swimmer plot. alloHCT, allogeneic hematopoietic cell transplantation; CR, complete response.

**Figure 3:**
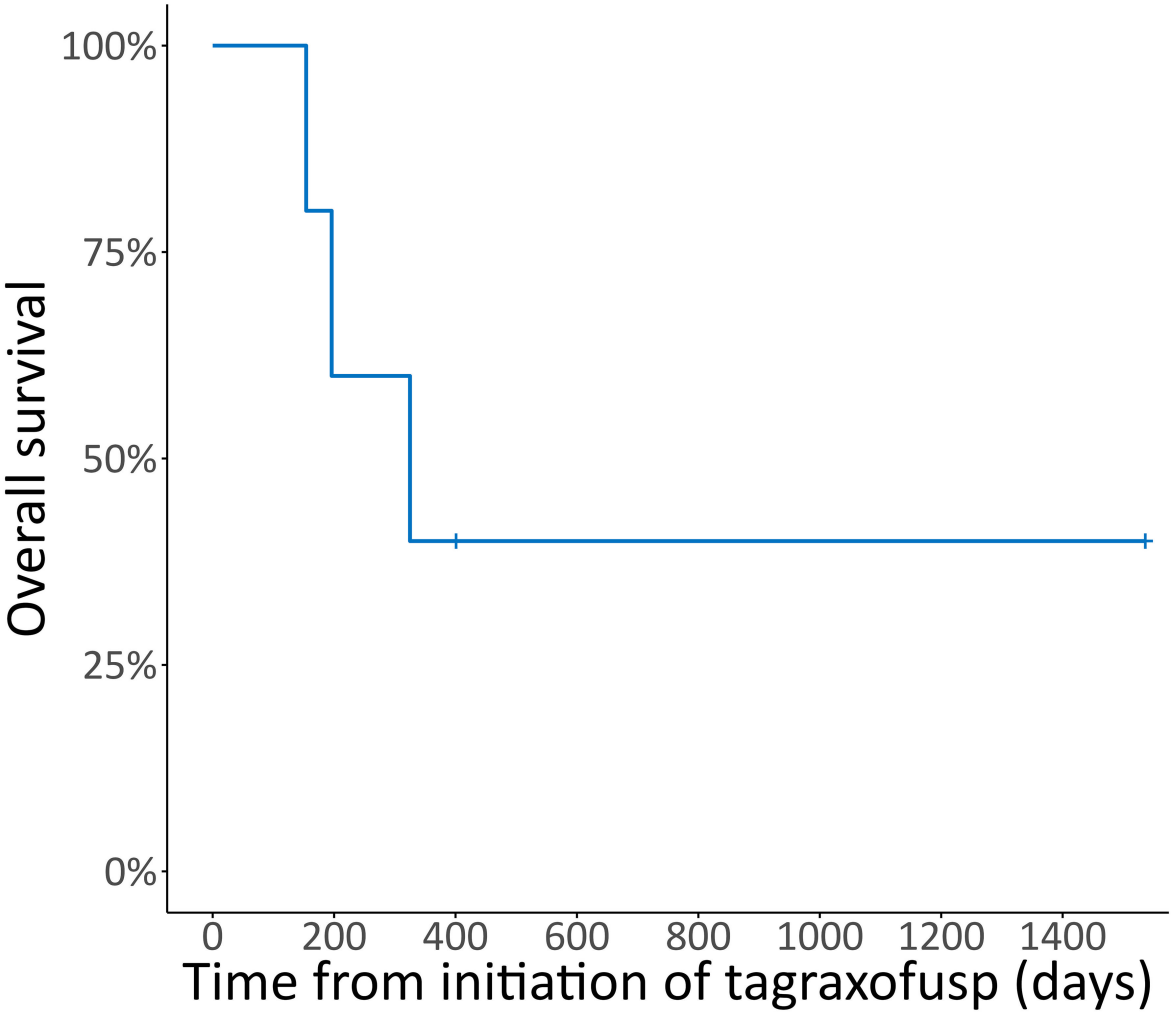
Overall survival. Kaplan-Meier plot for overall survival (OS) in % from initiation of tagraxofusp, median follow-up duration 10.6 months.

Two patients achieved a CR, one a PR, one patient had stable disease (SD) and one patient had progressive disease (PD) after treatment with tagraxofusp, with a response duration of 46, 11, 6 and 1 months, respectively, at a median follow-up of 11 months. One patient received alloHCT in CR (after only three doses of tagraxofusp) and is alive without relapse (**Figure 2**). The other patient in CR still continues tagraxofusp and has currently completed 13 cycles. Of the other three patients, one received alloHCT in PR, but unfortunately all three died from relapse or progressive disease 11, 5 and 4 months after starting treatment, respectively (**Table 2**, **Figure 3**). Relapse occurred in BM/skin and BM/lymph nodes/spleen respectively. Progressive disease was documented in the skin. Two patients received salvage treatment with venetoclax (Ven) at relapse, one in combination with Aza, but both responded poorly and died from clinically progressive disease one and three months after initiation of salvage treatment, respectively. The patient receiving Ven showed signs of progression with pancytopenia and BM infiltration of 95% one month after initiation. The patient treated with Aza/Ven achieved PR after one cycle with clearance of blasts in the PB but persistence of blasts in the BM. He developed neutropenia after cycle 1 and Aspergillus pneumonia. After cycle 2, therapy was stopped due to progression of pneumonia, clinical disease progression and delirium.

**Table 2:** Tagraxofusp treatment outcomes and adverse events (AEs).

	Tagraxofusp treatment						Outcomes					
#	Cycles	CLS	Thrombocyto-penia	Hepatotoxicity	Other AEs	alloHCT	Best response	Response duration	Relapse	Treatment for relapse	OS	Cause of death
1	5	–	–	–	AKI	–	SD	1 m	yes	Ven/Aza	4 m	PD
2	1	°2	°4	GPT °2, GOT °3	–	Yes	CR	46 m	no	–	50 m	–
3	13	°2	–	GPT °1, GOT °3	Cardiac decompensation, hypertension, NSTEMI, AKI, pneumonia	–	CR	11 m	no	–	13 m	–
4	2	°2	°1	GPT °1, GOT °1	fever, pneumonia	Yes	PR	6 m	yes	–	11 m	PD
5	2	–	–	–	–	–	PD	–	yes	radiotherapy, Ven	5 m	PD

alloHCT, allogeneic hematopoietic cell transplantation; AKI, acute kidney injury; Aza, azacitidine; CLS, capillary leak syndrome; CR, complete response; GPT, Glutamate pyruvate transaminase; GOT, Glutamate oxaloacetate transaminase; NSTEMI, non-ST-segment elevation myocardial infarction; PD, progressive disease; PR, partial response; SD, stable disease; Ven, venetoclax.

## 4 Discussion

In our cohort, the observed response and survival rates were lower than those reported in the clinical trials. However, our cohort showed higher age (79 vs. 70 years) and more comorbidities than the corresponding pivotal trial cohort (34). However, despite the advanced age and associated comorbidities, tagraxofusp demonstrated good tolerability, with only one patient experiencing third-degree CLS, leading to treatment discontinuation. Notably, this patient achieved a CR after three single doses of tagraxofusp, subsequently underwent alloHCT, and remained in ongoing remission for over 3 years, underscoring the significance of alloHCT for long-term survival.

One patient is alive and remains in CR under ongoing treatment with tagraxofusp. This patient was 84 years old at diagnosis, had a comorbidity index of 5, and would not have been eligible for either intensive chemotherapy or alloHCT. At the same time, he has tolerated tagraxofusp well and thus has benefited much from the availability of tagraxofusp. Apart from the above-mentioned clinical trials, real-world outcome data from tagraxofusp-treated BPDCN patients are limited to case reports and conference abstracts reporting early results (35).

We identified 13 case reports of tagraxofusp first-line treatment for BPDCN outside clinicals trials in the literature, with patients at a median age of 63 years (range 12-81), ten (76.9%) male and three female, who received a median of three cycles tagraxofusp (**Table 3**) (36–47). Of these, eight patients achieved a CR (61.5%), two a PR (15.4%) and two had stable disease, with a median response duration of 9 months for CR and 4 months for CR and PR combined. All eight patients with progression or relapse (61.5%) received salvage treatment, including Ven/Aza in four cases, decitabine/Ven in one case and intensive chemotherapy in four cases. Six patients received HCT (46.2%), five allogeneic and one autologous. Median OS of all 13 patients was 11.5 months, with nine of 13 patients being in CR at the time of the reporting. In this small group, OS > 12 months was associated with CR to tagraxofusp and HCT. Tagraxofusp was discontinued due to AEs in two cases (15.4%), with ≥ grade 3 CLS in five cases (38.5%) and other ≥ grade 3 AEs in four cases (30.8%). Interestingly, similar to one of our patients, one of the reported cases achieved a CR lasting 4 months after only three doses of tagraxofusp (45). However, reported cases were younger and had less comorbidities than our cohort. In addition, reporting bias needs to be accounted for.

**Table 3:** Reported cases of first-line tagraxofusp treatment for BPDCN.

Patient presentation					Tagraxofusp treatment				Outcomes								
#	Sex	Age	Comorbid diseases	Genetic alterations	Cycles	CLS °	Other AEs	HCT	Best response, duration	Relapse/PD	Salvage treatment, duration, best response	OS	Alive	Status	Author	Year	Country
1	m	70	None	NA	3	None	None	No	PD	PD	1. Pivekimab sunirine, 1 cycle, PD 2. Ven/Aza, 2 cycles, CR	≥35 m	Yes	CR	Azad et al.	2022	USA
2	f	81	Stroke, aHT, T2DM	ASXL1, IDH2, KRAS	2	4	foot gangrene, °4 TP. TAG discont’d.	No	CR, 20 m	No	None	20 m	Yes	CR	Sibai et al.	2022	Canada
3	m	37	None	NA	5	3	myocardial edema, °3 TP, °3 HT. Dose reduction.	alloHCT	CR, ≥ 18 m	No	None	≥ 1.5 y	Yes	CR	Mouhayar et al.	2021	USA
4	m	68	NA	ASXL1, TET2, SRSF2	2	≥ 3	≥ °3 HT. TAG discont’d.	No	≥ PR, < 2 m	Relapse	1. ICT, 4 m, PD 2. Ven/Aza, 1 cycle, PD, death	5 m	No	PD	Koerber et al.	2022	Germany
5	m	>75	NA	NA	6	None	NA	No	SD, 4 m	PD	Ven/Aza, 3 cycles, CR	≥ 6m	Yes	CR	Egger et al.	2021	USA/Ecuador
6	m	79	aHT, Hyperlipidemia	SF3B1, TET2, ZRSR2	8	3	Pancreatitis, AKI, hyperglycemia, NSTEMI, °4 TP, °3 HT. Dose reduction.	No	SD, 6 m	PD	Ven/Aza, 5 cycles, CR	11 m	Yes	CR	Samhouri et al.	2021	USA
7	f	38	NA	TP53, del 13q14, CEP9/9p21	1	2	HT, °4 TP, febrile neutropenia	alloHCT	CR, ≥ 9 m	No	None	≥ 10 m	Yes	CR	Acedo et al.	2021	Spain
8	m	52	None	NA	3	None	HT	autoHCT***	CR, < 3 m	CNS relapse	1. ICT 2 cycles; intrathecal chemotherapy 4 courses, CR 2. Consolidation with autoHCT, CR	≥ 12m	Yes	CR	Vangala et al.	2022	Germany
9	m	63	NA	NA	3	3	None	alloHCT	CR, ≥ 9 m	None	None	≥ 12m	Yes	CR	Massone et al.	2020	Italy
10	f	12	None	NRAS, Myb-PLEKH01	5	None	None	alloHCT***	PR < 3 m	PD	1. ICT 2 cycles, CR 2. Local radiotherapy 3. Consolidation with alloHCT, CR	≥ 17m	Yes	CR	Sun et al.	2018	USA
11	m	NA*	NA	None	1**	≥ 3	TAG discont’d.	No	CR, 4 m	Relapse	1. TAG 5 cycles, CR	12 m	Yes	Relapse	Sahin et al.	2024	USA
12	m	80	PV	del(20)(q11.2q13)	3	None	insomnia, anxiety, fever	No	possibly CR, < 4 m	NA	NA	4 m	No	NA	El Hussein et al.	2022	USA
13.	m	59	NA	TET2, ASXL1	3	NA	NA	alloHCT***	CR, < 1 y	Relapse	1. Deci/Ven 4 cycles, SD 2. Dara/Ven 4 cycles, CR 3. Hyper-CVAD 1 cycle followed by MTX/6.MP maintenance, CR 4. consolidation with alloHCT 5. IT therapy, CR	31 m	No	Relapse	Hu et al.	2024	USA

In case of incomplete data on follow-up and survival length, minimum duration was extrapolated from the description of treatments and indicated with a ≥ sign. * described as elderly. ** 3 doses. *** transplanted post relapse or progression. AE, adverse event; aHT, arterial hypertension; alloHCT, allogeneic hematopoietic cell transplantation; autoHCT, autologous HCT; AKI, acute kidney injury; Aza, azacitidine; BM, bone marrow; CLS, capillary leak syndrome; CNS, central nervous system; CR, complete response; Dara, daratumumab; Deci, decitabine; ICT, intensive chemotherapy; IT, intrathecal; LN, lymph nodes; M, months; NA, not available; NSTEMI, non-ST-segment elevation myocardial infarction; PD, progressive disease; PR, partial response; PV, polycythemia vera; SD, stable disease; T2DM, type 2 diabetes mellitus; Ven, venetoclax; Y, year.

AlloHCT was shown to improve OS in patients with BPDCN. Retrospective analyses by Murphy et al. in elderly or frail patients with BPDCN and alloHCT indicate that mostly reduced intensity conditioning was used, and that lack of CR and age over 60 may associate with worse outcome, however also this subset of patients shows OS rates of 40-50% after alloHCT (25).

Long-term alloHCT outcomes after first-line treatment with tagraxofusp have not been analyzed in larger cohorts as of yet, but the available case reports and our series suggest that all eligible patients should be considered for alloHCT. In the report by Yun et al., treatment with tagraxofusp was shown to induce CR in 6 of 12 treated patients. Of these, three subsequently were sufficiently fit to receive alloHCT (48). In our series, the patients who received alloHCT were 63 and 68 years old. Both received conditioning in analogy to other elderly patients treated at our center and showed comparable tolerability to these age-matched patients. In another report, Bashir et al. report on six patients with BPDCN treated with tagraxofusp followed by alloHCT, three of which received hyper-CVAD additionally to tagraxofusp. Interestingly, treatment intensification with hyper-CVAD did not improve survival when compared to tagraxofusp alone (median OS 29.5 months versus 32.9 months) (49).

Unfortunately, limited effective therapies are available beyond tagraxofusp, particularly for refractory or relapsed disease (50). Adaptive resistance to tagraxofusp has been shown to be mediated by the modification of the target of diphtheria toxin (51), independent of CD123 downregulation, and may be targeted by Aza (27, 52). In addition, tagraxofusp-resistant cells have been demonstrated to exhibit increased dependence on BCL2 (53). Although our findings did not show favorable responses to salvage treatment with Aza and/or Ven, these agents also hold promise for post-tagraxofusp relapse salvage treatment, as indicated by several case reports (36, 40, 41).

The combination of tagraxofusp with Aza and/or Ven is currently being evaluated in a phase 1b trial for BPDCN and AML (NCT03113643), with good tolerability and a promising response rate of 69% already reported for adverse risk AML patients within the study (53). In addition, tagraxofusp is being evaluated as first-line treatment for BPDCN in combination with chemotherapy and Ven (NCT04216524), for post-HCT relapse and maintenance (NCT04317781), and in pediatric patients with R/R BPDCN. Other treatments that are currently being evaluated in clinical trials include CD123-targeted adoptive CAR T or CAR NK cells (NCT04318678, NCT04230265, NCT06006403), an anti-CD123 antibody-drug conjugate (NCT03386513), and Ven for R/R BPDCN or patients ineligible for intensive treatment (NCT03485547).

Currently, tagraxofusp treatment is generally continued until progression or development of intolerable adverse effects. Future studies might be able to define criteria for treatment discontinuation and re-exposure. In view of potentially lower efficacy in elderly patients and high treatment cost, future comparisons are needed between tagraxofusp and other agents, and include an appraisal of patient-reported outcome (PRO) or quality of life measures especially in elderly and unfit patients. The findings from our cohort underscore the need for continued research and the development of effective therapies, especially for elderly patients with BPDCN and patients with relapsed or refractory disease post-tagraxofusp. However, although this series and most published cases report on older patients, two case series indicate promising response and survival rates of tagraxofusp in pediatric and adolescent/young adult BPDCN patients (44, 54). To evaluate safety and efficacy in a real-world setting, a prospective registry has been started (EudraCT: 2021-001684-24).

We report real-world data on the efficacy and safety of tagraxofusp as first-line treatment for BPDCN in a cohort of five patients, some elderly and with significant comorbidities, and discuss further 13 reported real-world cases. Our findings generally support the use of tagraxofusp for first-line treatment of BPDCN. Furthermore, our series suggests that tagraxofusp can safely be used in selected elderly and medically non-fit patients with BPDCN, if appropriate monitoring and standards for the screening and intervention in case of CLS development are established. The study also emphasizes the potential of tagraxofusp in the context of alloHCT and the importance of proactive CNS screening and management. These insights contribute to the growing body of evidence on the use of tagraxofusp in the treatment of BPDCN and provide valuable clinical considerations for managing this challenging disease.

## Data availability statement

The datasets presented in this article are not readily available because data are available on bases of institutional review board-approved data request in alignment with applicable data protection regulations. Requests to access the datasets should be directed to jan.schroeder@med.uni-tuebingen.de.

## Ethics statement

The studies involving humans were approved by the Ethics Committee at the Medical Faculty of the Eberhard Karls University and at the University Hospital of Tübingen. The studies were conducted in accordance with the local legislation and institutional requirements. Written informed consent for participation was not required from the participants or the participants’ legal guardians/next of kin in accordance with the national legislation and institutional requirements. Written informed consent was not obtained from the individual(s) for the publication of any potentially identifiable images or data included in this article because the patient had died at the time of manuscript preparation. An image of a typical cutaneous manifestation was included with appropriate cropping to preclude identification of the respective patient.

## Author contributions

PF: Data curation, Formal analysis, Investigation, Methodology, Resources, Visualization, Writing – original draft. JCS: Conceptualization, Data curation, Formal analysis, Investigation, Methodology, Resources, Visualization, Writing – original draft, Writing – review & editing. LM: Resources, Visualization, Writing – review & editing. LH: Resources, Writing – review & editing. AR: Formal analysis, Writing – review & editing. WV: Writing – review & editing, Conceptualization, Supervision. CL: Conceptualization, Supervision, Writing – review & editing, Project administration, Validation. SW: Writing – review & editing.
